# Aberrant DNA methylation of cancer-related genes in giant breast fibroadenoma: a case report

**DOI:** 10.1186/1752-1947-5-516

**Published:** 2011-10-18

**Authors:** Diego M Marzese, Francisco E Gago, Javier I Orozco, Olga M Tello, María Roqué, Laura M Vargas-Roig

**Affiliations:** 1 Cellular and Molecular Laboratory, IHEM-CCT-CONICET, Parque General San Martín s/n, CP 5500, Mendoza, Argentina; 2 School of Medical Sciences, National University of Cuyo, Parque General San Martín s/n, CP 5500, Mendoza, Argentina; 3 Gineco-Mamario Institute, San Lorenzo 536, CP 5500, Mendoza, Argentina; 4 Tumor Biology Laboratory, IMBECU-CCT-CONICET, Avda Adrian Ruiz Leal s/n, Parque General San Martín, CP 5500, Mendoza, Argentina

## Abstract

**Introduction:**

Giant fibroadenoma is an uncommon variant of benign breast lesions. Aberrant methylation of CpG islands in promoter regions is known to be involved in the silencing of genes (for example, tumor-suppressor genes) and appears to be an early event in the etiology of breast carcinogenesis. Only hypermethylation of p16INK4a has been reported in non-giant breast fibroadenoma. In this particular case, there are no previously published data on epigenetic alterations in giant fibroadenomas. Our previous results, based on the analysis of 49 cancer-related CpG islands have confirmed that the aberrant methylation is specific to malignant breast tumors and that it is completely absent in normal breast tissue and breast fibroadenomas.

**Case presentation:**

A 13-year-old Hispanic girl was referred after she had noted a progressive development of a mass in her left breast. On physical examination, a 10 × 10 cm lump was detected and axillary lymph nodes were not enlarged. After surgical removal the lump was diagnosed as a giant fibroadenoma. Because of the high growth rate of this benign tumor, we decided to analyze the methylation status of 49 CpG islands related to cell growth control. We have identified the methylation of five cancer-related CpG islands in the giant fibroadenoma tissue: ESR1, MGMT, WT-1, BRCA2 and CD44.

**Conclusion:**

In this case report we show for the first time the methylation analysis of a giant fibroadenoma. The detection of methylation of these five cancer-related regions indicates substantial epigenomic differences with non-giant fibroadenomas. Epigenetic alterations could explain the higher growth rate of this tumor. Our data contribute to the growing knowledge of aberrant methylation in breast diseases. In this particular case, there exist no previous data regarding the role of methylation in giant fibroadenomas, considered by definition as a benign breast lesion.

## Introduction

Fibroadenoma represents the most frequent breast lesion in adolescents and young women with the giant fibroadenoma (GF) being an uncommon variant. GFs, which occur mostly in adolescent girls, are characterized by their large size (more than 5 cm). They are encapsulated masses and generally asymptomatic. Their rapid growth (between two and five months) is associated with skin congestion and ocasionally ulceration. It is thought that increased estrogen receptor sensitivity is responsible for the etiology of GF [1].

Aberrant methylation of CpG islands (CpGIs) in promoter regions is known to be involved in the silencing of tumor-suppressor genes, steroid receptors, cell adhesion molecules and cell cycle regulator genes and appears to be an early event in the etiology of breast carcinogenesis [2]. The aberrant methylation of cell cycle regulator genes leads to a higher proliferation rate [3].

Our previous results, based on the analysis of 49 cancer-related CpGIs, have confirmed that the aberrant methylation is specific to malignant breast tumors and that it is completely absent in normal breast tissue and breast fibroadenomas [4]. Other authors have reported aberrant methylation of p16INK4a not only in malignant breast lesions but also in fibroadenoma and normal mammary tissues [5]. There are no previous data of epigenetic alterations in giant fibroadenomas. The established precursors of breast carcinoma are atypical ductal hyperplasia, ductal carcinoma *in situ *, and lobular neoplasia. The malignant transformation of a fibroadenoma is a rare event, with about 100 cases reported in the world literature. Despite this fact we decided to analyze the methylation status of a GF which is a rapidly growing benign breast lesion [6], because the methylation of the analyzed genes is associated with a greater capacity for cell growth [3].

## Case presentation

A 13-year-old Hispanic girl was referred after she had noted the progressive development of a mass in her left breast. On physical examination, a 10 × 10 cm lump was detected. Her axillary lymph nodes were not enlarged. Surgery was performed and a GF was removed. At present, with a follow-up of three years, both breasts are symmetrical, normally developed, and no signs of recurrence have been detected at clinical evaluations.

Methylation-specific multiplex ligation-dependent probe amplification (MS-MLPA) assay was performed on the DNA obtained from the GF to study the methylation status of the 49 CpGIs (Table 1). We have previously analyzed these regions in invasive ductal carcinomas, breast fibroadenomas and normal mammary tissue [4]. The MS-MLPA Kits ME001 and ME002 were used according to the manufacturer's recommendations (MRC-Holland, Amsterdam, Netherlands) with minimal modifications [4].

**Table 1:** CpG Islands analyzed

	Gene	Region		Gene	Region		Gene	Region		Gene	Region		Gene	Region
1	APC	-21 bp	11	CDH13	186 bp	21	IGSF4	-56 bp	31	p73	+258 bp	41	RASSF1	+46 bp

2	ATM	+309 bp	12	CHFR	-103 bp	22	IGSF4	-294 bp	32	p73	+25 bp	42	RB1	-226 bp

3	ATM	+138 bp	13	CHFR	-96bp	23	MGMT	-463 bp	33	PAX5	-120 bp	43	RB1	-449 bp

4	BRCA1	-20bp	14	DAPK1	+527 bp	24	MLH1	+55 bp	34	PAX6	-52 bp	44	STK11	+416 bp

5	BRCA1	+86bp	15	ESR1	+244 bp	25	MLH1	-320 bp	35	PTEN	-813 bp	45	THBS1	-791 bp

6	BRCA2	+221 bp	16	FHIT	+225 bp	26	p15	+473 bp	36	PTEN	-66 bp	46	TIMP3	+1019 bp

7	BRCA2	+138 bp	17	GATA5	+271 bp	27	p16	-817 bp	37	PYCARD	+437 bp	47	VHL	+115 bp

8	CASP8	+291bp	18	GSTP1	+148 bp	28	p16	+200 bp	38	RARβ	-357 bp	48	VHL	-3 bp

9	CD44	+411 bp	19	GSTP1	+468 bp	29	P27	+307 bp	39	RARβ	-180 bp	49	WT1	-210 bp

10	CD44	+28 bp	20	HIC1	-6 bp	30	P53	+100 bp	40	RASSF1	-136 bp			

The table shows the 49 genomic regions tested during the study. Positive and negative signs are related to the transcription start base pair.

The immunohistochemical procedure was performed as reported previously using the monoclonal antibody clone ER88 (Biogenex, CA, USA) against estrogen receptor alpha protein [7].

**Figure 1:**
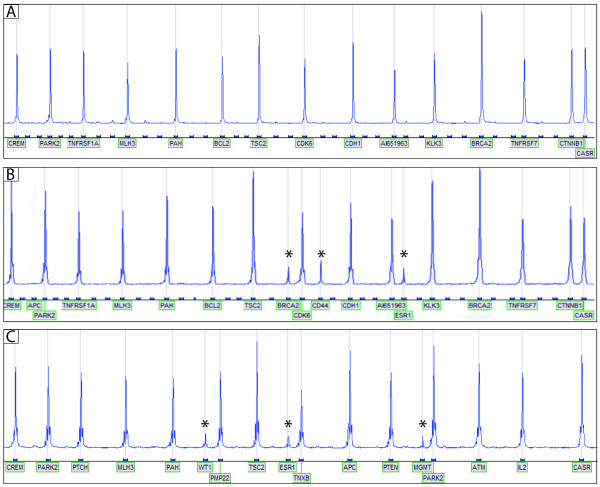
**Detection of aberrant DNA methylation in the giant fibroadenoma**. **A**: MS-MLPA analysis of DNA isolated from non-giant fibroadenoma. None of the analyzed regions are methylated. Only the PCR products from control probes are detected. **B **and **C**: MS-MLPA analysis of DNA isolated from the giant fibroadenoma. The methylation specific peaks are marked with an asterisk (*). Panel **B **shows the presence of methylation in BRCA2, CD44 and ESR1 genes and panel **C **shows the methylation of WT1, ESR1 and MGMT genes.

We have detected aberrant methylation in five cancer-related CpGIs, that is estrogen receptor-α [ESR1 (+244bp)], O6-methylguanine-DNA methyltransferase [MGMT (-463bp)], Wilms' Tumor-1 [WT-1 (-146bp)], Breast Cancer 2 [BRCA2 (+138bp)] and Hermen Antigen [CD44 (+28bp)] (Figure 1). As a control we have analyzed six normal breast tissues and three breast fibroadenomas from 21-, 23- and 29-year-old patients. None of these samples showed methylation in any of the 49 CpGIs.

**Figure 2:**
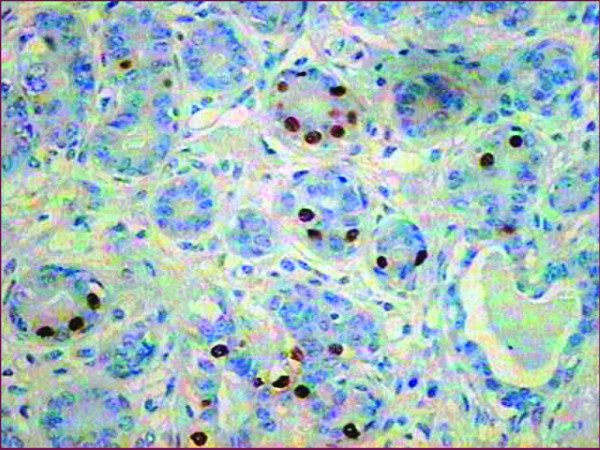
**Immunostaining of ERα protein**. The figure shows the staining in the nuclei of a few epithelial cells of the giant fibroadenoma (400x).

In order to evaluate the effect of the aberrant methylation on the level of protein expression in the fibroadenoma, we investigated the expression of ERα protein observing a moderate intensity in only 15% of the fibroadenoma epithelial cells (Figure 2).

## Discussion

To the best of our knowledge, the only reported aberrant methylation in fibroadenomas is in gene p16INK4a. Our previous results analyzing a 49-gene regions panel which does not include the same reported CpGI of p16INK4a- have not revealed aberrant methylation in benign breast lesions [4,5].

Our finding of five aberrant methylated regions in the reported GF suggests that this type of fibroadenoma presents a different etiology than other benign breast lesions, at least regarding the methylation profile.

In invasive breast tumors we have detected from two to 23 aberrantly methylated cancer-related regions, which indicates that five affected CpGIs is not a high number for a breast carcinoma (unpublished data). The surprising novelty, however, is that this finding occurs in a benign lesion.

These five aberrant methylated genes play diverse functions in the cell: DNA reparation (MGMT and BRCA2), cell cycle control (BRCA2, WT1), proliferation (WT1, ESR1) and cell adhesion (CD44). The methylation of three of them (ESR1, MGMT and WT1) has been widely reported in breast tumors [2,4,8]. Methylation of WT1 has not been found in normal tissue [9]. Previous studies have reported the methylation of BRCA2 in breast tumor but to the best of our knowledge, our study is the first to find methylated BRCA2 in benign breast disease [10]. Regarding gene CD44, as far as we know, its methylation status has not been reported in mammary tissue before, even though new evidence suggests its methylation in the breast cancer cell line MCF7 [11]. Methylation of the ESR1 promoter and its first exon has been observed to be correlated with loss of the expression of ERα receptor, even though some breast cancer specimens maintain its expression (ER+) [12-14]. Tests based on ERα staining in fibroadenoma reveal a pronounced heterogeneity (range between 1% and 85%) showing no age correlation [15]. Our specimen expresses 15% of ERα protein, which is considered low. Even though we are not able to establish the percentage of methylated ESR1 genes in the GF, given its heterogeneity, this low protein expression is in accordance with the determined methylated gene profile. The methylation of these five regions could be responsible in part for the high growth rate present in the analyzed GF.

## Conclusions

Our data contribute to the growing knowledge of aberrant methylation in breast diseases. In this particular case there were no previously published data regarding the role of methylation in GFs, considered by definition to be a benign breast lesion. These findings should be taken into account to evaluate whether it is associated with the different etiology of non-GFs and GFs. Further studies will be necessary to draw more definitive conclusions about the meaning of the methylation de-regulation in this type of disease.

## Abbreviations

BRCA2: Breast Cancer 2; CD44: Hermen Antigen; CpGIs: CpG islands; ERα: estrogen receptor α protein; ESR1: estrogen receptor-α; GF: giant fibroadenoma; MGMT: O6-methylguanine-DNA methyltransferase; MS-MLPA: Methylation-specific multiplex ligation-dependent probe amplification; p16INK4a: Cyclin-dependent kinase inhibitor 2A; WT-1: Wilms' Tumor-1

## Consent

Written informed consent was obtained from the patient's next-of-kin for publication of this case report and any accompanying images. A copy of the written consent is available for review by the Editor-in-Chief of this journal.

The study was approved by the Bioethics Committee of the School of Medical Sciences, National University of Cuyo, Mendoza, Argentina.

## Competing interests

The authors declare that they have no competing interests.

## Authors' contributions

DMM performed the methylation study and revised the manuscript critically. FEG participated in the study design with JO. OT carried out the pathological studies. MR participated in interpretation of data and revised the manuscript critically. LMV-R designed the study and wrote the manuscript. All the authors discussed the results and read and approved the final manuscript.
